# TET2 Mutation and High miR-22 Expression as Biomarkers to Predict Clinical Outcome in Myelodysplastic Syndrome Patients Treated with Hypomethylating Therapy

**DOI:** 10.3390/cimb43020065

**Published:** 2021-08-05

**Authors:** Jina Yun, Young Sok Ji, Geum Ha Jang, Sung Hee Lim, Se Hyung Kim, Chan Kyu Kim, Sang Byung Bae, Jong Ho Won, Seong Kyu Park

**Affiliations:** 1 Department of Internal Medicine, Division of Hemato-Oncology, Soonchunhyang University Bucheon Hospital, 170 Jomaru-ro, Wonmi-gu, Bucheon-si 14584, Gyeonggi-do, Korea; 19983233@schmc.ac.kr (J.Y.); youngsokji@gmail.com (Y.S.J.); pleiades75@hanmail.net (G.H.J.); sungheelim@schmc.ac.kr (S.H.L.); shkim@schmc.ac.kr (S.H.K.); md53097@schmc.ac.kr (C.K.K.); 2 Department of Internal Medicine, Division of Hemato-Oncology, Soonchunhyang University Cheonan Hospital, 31, Suncheonhyang 6-gil, Dongnam-gu, Cheonan-si 31151, Chungcheongnam-do, Korea; baesan@schmc.ac.kr; 3 Department of Internal Medicine, Division of Hemato-Oncology, Soonchunhyang University Seoul Hospital, 59, Daesagwan-ro, Yongsan-gu, Seoul 04401, Korea; jhwon@schmc.ac.kr

**Keywords:** TET2, miR-22, hypomethylating therapy, myelodysplastic syndrome (MDS), hypermethylation, cytogenetic abnormality

## Abstract

Tet methylcytosine dioxygenase 2 (TET2) is one of the most frequently mutated genes in myelodysplastic syndrome (MDS). TET2 is known to involve a demethylation process, and the loss of TET2 is thought to cause DNA hypermethylation. Loss of TET2 function is known to be caused by genetic mutations and miRNA, such as miR-22. We analyzed 41 MDS patients receiving hypomethylating therapy (HMT) to assess whether TET2 mutation status and miR-22 expression status were associated with their clinical characteristics and treatment outcomes. Responsiveness to HMT was not affected by both TET2 mutation (odds ratio (OR) 0.900, *p* = 0.909) and high miR-22 expression (OR 1.548, *p* = 0.631). There was a tendency for TET2 mutation to be associated with lower-risk disease based on IPSS (Gamma = −0.674, *p* = 0.073), lower leukemic transformation (OR 0.170, *p* = 0.040) and longer survival (Hazard ratio 0.354, *p* = 0.059). Although high miR-22 expression also showed a similar tendency, this tendency was weaker than that of TET2 mutation. In summary, the loss of TET2 function, including both TET2 mutation and high miR-22 expression, was not a good biomarker for predicting the response to HMT but may be associated with lower-risk disease based on IPSS, lower leukemic transformation and longer survival.

## 1. Introduction

Myelodysplastic syndromes (MDS) are clonal stem cell malignancies characterized by cytopenia, inefficient hematopoiesis, and dysplasia in one or more myeloid cell lineages and increased risk of development of acute myeloid leukemia (AML) [1]. In Korea, MDS is the third most common myeloid malignancy [2]. Until now, many types of genetic alteration, including chromosomal abnormalities and somatic mutations, have been found in MDS, some of which may be driver mutations underlying the development of MDS [3]. The driver genes frequ ently mutated in MDS include DNA methylation, chromatin modification and other discrete functional pathways. Specifically, genes involved in DNA methylation and chromatin modification are mutated in about 60–70% of MDS cases. Among these genes, Tet methylcytosine dioxygenase 2 (TET2) and DNA methyltransferase 3A (DNMT3A) are known to be the predominant targets. Mutations of these genes are known to be related to age-related clonal hematopoiesis, suggesting their role as founder mutations in the early development of MDS.

Functionally, mutations of genes involved in epigenetic modification induce epigenetic dysregulation, such as aberrant DNA hypermethylation in MDS [1]. Aberrant DNA hypermethylation is thought to contribute to clonal evolution in MDS by silencing the expression of tumor suppressor genes (TSGs). Therefore, hypomethylating agents, such as azacitidine (AZA) and decitabine (DAC), have been used to reverse aberrant DNA hypermethylation in MDS. As DNMT1 inhibitors, hypomethylating agents are expected to induce transient DNA hypomethylation to allow re-expression of TSGs, triggering the death or differentiation of MDS cells [4]. Although hypomethylating therapy (HMT) using AZA or DAC has emerged as a common frontline therapy for patients with MDS contraindicated for intensive chemotherapy, its efficacy is not robust in the majority of patients [5]. In addition, another disadvantage of HMT is that the response to these agents is usually seen after the fourth or sixth cycle of treatment [6]. Therefore, the expert panel recommends that patients should be treated initially for a minimum of four to six cycles in the absence of disease progression or unacceptable toxicity [7]. Under these circumstances, several studies have been conducted to identify markers for predicting patients who may benefit from HMT. For example, Kim et al. suggested that bone marrow (BM) microvessel density, one of the biomarkers of angiogenesis, may predict responsiveness to HMT [8].

TET2, which is involved in epigenetic modification, is one of the most frequently mutated genes in MDS [9]. Similar to other TET proteins, such as TET1 and TET3, TET2 is known to catalyze the oxidation of 5-methylcytosine into 5-hydroxymethylcytosine and then into 5-formylcytosine and 5-carboxylcytosine, which are repaired into unmethylated cytosines via active DNA demethylation in TET/TDG (thymine–DNA–glycosylase)/BER (base excision repair)-dependent pathway. In hematologic diseases, loss of TET2 may cause DNA hypermethylation in enhancer-like regions but not in CpG sites in promoters [10,11]. Furthermore, Rasmussen et al. reported that the loss of TET2 in hematopoietic cells resulted in the DNA hypermethylation of active enhancers and induction of leukemogenesis [10].

Loss of TET2 function is caused by genetic mutations, miRNA interference, and metabolites, such as 2-hydroxyglutarate. Cheng et al. identified > 30 miRNAs that inhibit TET2 expression [12]. They also demonstrated that forced expression of TET2-targeting miRNAs in vivo disrupts normal hematopoiesis, leading to hematopoietic expansion and/or myeloid differentiation bias. In addition, Song et al. reported that the oncogenic miR-22 also targets the TET2 to promote hematopoietic stem cell self-renewal and transformation [13].

**Figure 1:**
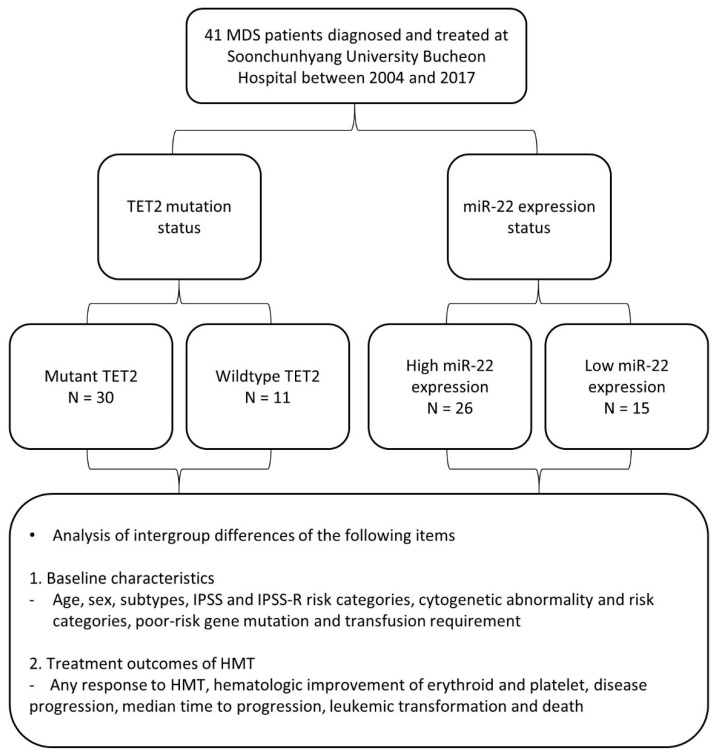
Flow chart of this retrospective study. Abbreviations: HMT, hypomethylating therapy; MDS, myelodysplastic syndrome; TET2, Tet methylcytosine dioxygenase 2; IPSS, International Prognostic Scoring System; IPSS-R, Revised International Prognostic Scoring System.

Theoretically, the loss of TET2 is a good candidate for HMT. Indeed, some studies reported that TET2 mutation could predict the response to HMT [14,15]. In this study, we evaluated whether TET2 mutation and miRNA-22 expression are predictive markers of response to HMT. We evaluated TET2 mutation status and miRNA-22 expression level in BM cells of MDS patients and assessed their relationship with baseline characteristics and treatment outcomes of HMT (Figure 1).

## 2. Materials and Methods

### 2.1. Patient Characteristics and Sample Preparations

A total of 41 MDS patients diagnosed and treated at Soonchunhyang University Bucheon Hospital between 2004 and 2017 were included in this study. Written informed consent was obtained from all patients. BM aspirates were collected from these patients. BM aspirates underwent RBC lysis (BD Pharm Lyse™ lysing solution, BD Bioscience, San Jose, CA, USA) and were cryopreserved for storage at the Soonchunhyang University Bucheon Hospital Clinical Laboratory, a tissue repository approved by the local Institutional Review Board.

All laboratory and clinical data were obtained at the time of diagnosis. MDS subtypes were defined according to the WHO classification [16]. Based on the IPSS (International Prognostic Scoring System) [17] and IPSS-R (Revised International Prognostic Scoring System) [18], patients were divided into lower-risk disease (IPSS low, intermediate-1; IPSS-R very low, low, intermediate) and higher-risk disease (IPSS intermediate-2, high; IPSS-R high, very high). The cytogenetic risk categories for MDS were defined according to IPSS-R. The cytogenetic risk categories were divided into lower-risk karyotype (IPSS-R very good, good) and higher-risk karyotype (IPSS-R intermediate, poor, very poor). All patients received HMT using either AZA or DEC. Response to HMT was assessed using the International Working Group response criteria revised in 2006 [19]. Patients showing either a complete response, partial response or hematologic improvement were considered as ‘responders’. Hematologic improvement was assessed based on erythroid and platelet responses.

### 2.2. Next-Generation Sequencing (NGS) for the Detection of Mutations in TET2 and Other Genes

DNA exon sequences in TET2, SF3B1, SRSF2, U2AF1, ZRSR2, IDH1, IDH2, DNMT3A, EZH2, ASXL1, SETBP1, TP53, PHF6, RUNX1, ETV6, CBL1, NRAS, KIT, JAK2, MPL and NPM1 were determined from cells in BM aspirates using NGS assay. Briefly, genomic DNA of BM aspirate cells was extracted using a DNeasy Blood & Tissue kit (Qiagen, Valencia, CA, USA). Random DNA fragments were generated from genomic DNA samples, and a barcode sequence and an adapter sequence for each sample were attached to each other to generate a library. Target enrichment process for TET2 and other genes was performed via oligonucleotide hybridization-based target capture. The flow cell was mounted on the large-scale sequencing equipment MiSeq system (Illumina, San Diego, CA, USA), followed by dispensing and sequencing of the sample library and reagents. The lower limit of detection of the assay was 5%, with a minimum depth of coverage of 500×. After acquiring the raw data (FASTQ file) from the equipment, the data was checked for quality and mapped to obtain the BAM file from which the mapping information was analyzed, and the VCF file was obtained through the variant calling process. Finally, the variant file was obtained via variant annotation and filtering to interpret the mutation status of TET2 and other genes. Variants were excluded from statistical analysis if they were labeled as benign/likely benign in ClinVar and/or COSMIC databases. Poor-risk gene mutation was defined by the presence of significant gene mutations, which are associated with a poor prognosis. Based on NCCN guidelines for MDS version 1.2021 [20] and the study of Hou et al. [21], genes such as TP53, EZH2, ASXL1, DNMT3A, SRSF2, IDH2 and NPM1 were designated as poor-risk genes.

### 2.3. Detection of TET2 Deletion Using Fluorescence In Situ Hybridization (FISH)

TET2 deletion was assessed using FISH. Briefly, FISH was conducted with the interphase nuclei of BM aspirate cells using BACs covering the TET2 gene (RP11-351K6 and RP11-16G16; BlueGnome, Cambridge, UK) and a commercially available TET2 probe (MetaSystems; Altlussheim, Germany). Both assays were validated using samples with TET2 deletions proven by SNP 6.0 DNA microarray analysis (Affymetrix, Santa Clara, CA, USA). Hybridization spots were obtained according to the manufacturer’s recommendations, and 200 nuclei were examined per sample. FISH analyses were automatically performed. TET2 deletion was considered a significant TET2 mutation.

### 2.4. Real-Time Quantitative PCR of miR-22

miRNAs were extracted from BM aspirate cells using a miRNeasy Mini kit (Qiagen, Valencia, CA, USA). RNA concentration was determined using a NanoDropTM 2000/2000c spectrophotometer (ThermoFisher Scientific, Rockford, IL, USA). cDNA was obtained with a Superscript II RT kit (Applied Biosystems, Waltham, MA, USA) and a miScript II RT Kit (Qiagen, Valencia, CA, USA).

The level of miR-22-3p expression was measured with 7500 Real-Time PCR System (Applied Biosystems, Waltham, MA, USA) using PowerUp SYBR Green Master Mix (Applied Biosystems, Waltham, MA, USA) and a miScript SYBR^®^ Green PCR Kit (Qiagen, Valencia, CA, USA). Each value was normalized with RNU6B as an internal control of miR-22 expression. Patients were divided into two groups based on normalized miR-22 expression: ≥ 2.5 was considered high; <2.5 was considered low. The cut-off value was 2.5 based on the study results of Song et al. [11]. In this study, the reduction of TET2 expression by miR-22 was confirmed in both K562 and U937 cell lines, in which the value of miR-22 expression level normalized by RNU6B was about 2.5 to 3.5. Oligonucleotide sequences for miR-22 and RNU6B were obtained from miScript primer assays (Qiagen, Valencia, CA, USA). The PCR conditions were set as follows: 50 °C for 2 min (UDG activation step), 95 °C for 2 min, 40 cycles of 95 °C for 15 s, 60 °C for 60 s, and 95 °C for 15 min, 40 cycles of 94 °C for 15 s, 55 °C for 30 s, and 70 °C for 30 s.

### 2.5. Statistical Analysis

Statistical differences in demographic and clinical characteristics according to TET2 mutation status and miR-22 expression status were evaluated using the Chi-square test, Fisher’s exact test or linear-by-linear association test for categorical variables and Student’s *t*-test or Wilcoxon’s rank-sum test for continuous variables. Before the *t*-test, a Shapiro–Wilk test for normality and Levene’s homogeneity of variance test was conducted. The Chi-square test or Fisher’s exact test was also used to compare treatment outcomes, including response rate to HMT, disease progression rate, leukemic transformation and mortality, except for median time to progression. Median time to progression was calculated with the Kaplan–Meier method using intention-to-treat (ITT) analysis and was compared using a log-rank test. Univariate logistic regression analysis was conducted to identify clinical factors that affected responsiveness to HMT and leukemic transformation. Univariate Cox proportional hazards regression analysis was performed to analyze the association between clinical factors and overall survival (OS). Overall survival probabilities and curves were estimated with the Kaplan–Meier method using ITT analysis and were compared using a log-rank test. The strength of correlation between two categorical variables was analyzed using Goodman–Kruskal’s gamma. *p* < 0.05 was considered statistically significant. All statistical analyses were performed using IBM SPSS statistics version 26 for Windows.

## 3. Results

### 3.1. Comparison of Baseline Characteristics and Treatment Outcomes According to TET2 Mutation Status

Of 41 MDS patients, 30 had wild-type (WT) TET2, and 11 had mutant TET2. Baseline characteristics of patients according to TET2 mutation status are listed in Table 1. The median age and sex ratios were not significantly different between the two groups. The distribution of MDS subtypes was not different between the two groups. The distribution of IPSS and IPSS-R risk categories differed significantly between the two groups (*p*-values = 0.039 and 0.009, respectively). The patients in the mutant TET2 group tended to have lower-risk disease based on IPSS and IPSS-R compared with those in the WT TET2 group (*p*-values = 0.075 and 0.006, respectively). Cytogenetic abnormality tended to be less frequent in the mutant TET2 group than in the WT TET2 group but was not statistically significant (18.2% vs. 43.3%, *p*-value = 0.168). Interestingly, while all patients in the mutant TET2 group exhibited lower-risk karyotype, some patients in the WT TET2 group showed higher-risk karyotype. However, this difference was not statistically significant (*p*-value = 0.083). The presence of poor-risk gene mutation was not different between the two groups (43.3% vs. 36.4%, *p*-value = 0.736). The proportion of patients requiring transfusion was not different between the two groups (86.7% vs. 72.7%, *p*-value = 0.361).

The association of TET2 mutation status with various treatment outcomes of HMT was analyzed using the Chi-square test or Fisher’s exact test and the Kaplan–Meier method (Table 2). The rate of any response to HMT was 83.3% (25/30) in the WT TET2 group and 81.8% (9/11) in the mutant TET2 groups (*p*-value = 1.000). Specifically, both erythroid and platelet response rates were not significantly different between the two group (*p*-values = 0.491 and 0.272, respectively). Disease progression was more frequently observed in the WT TET2 group than in the mutant TET2 group (63.3% vs. 27.3%, *p* = 0.040). The median time to progression of the mutant TET2 group was significantly longer than that of the WT TET2 group (49.8 months vs. 19.3 months, *p* = 0.007). Leukemic transformation was also more frequent in the WT TET2 group than in the mutant TET2 group (56.7% vs. 18.2%, *p* = 0.029). With a median follow-up time of 27.1 months (range, 7.5 to 83.5 months) in the WT TET2 group and 27.2 months (range, 8.7 to 95.9 months) in the mutant TET2 groups, the mortality rate was 76.7% (23/30) in the WT TET2 group and 36.4% (4/11) in the mutant TET2 group (*p*-value = 0.026).

### 3.2. Comparison of Baseline Characteristics and Treatment Outcomes of MDS Patients According to miR-22 Expression Status

Of 41 MDS patients, miR-22 expression was low in 26 and high in 15 cases. The baseline characteristics of patients according to miR-22 expression status are listed in Table 3. The median age and sex ratios were not significantly different between the two groups. The subtypes of MDS varied in both groups (*p*-value = 0.061).

The distribution of IPSS risk categories was not significantly different between the two groups (*p*-value = 0.149). Although the patients in the high miR-22 expression group tended to have lower-risk disease based on IPSS compared with those in the low miR-22 expression group, it was not statistically significant (*p*-value = 0.091). The distribution of IPSS-R risk categories did not vary significantly between the two groups (*p*-value = 0.429). Cytogenetic abnormality was not significantly different between the two groups (34.6% vs. 40.0%, *p*-value = 0.730). The distribution of cytogenetic risk categories was also not significantly different between the two groups (*p*-value = 0.757). The prevalence of poor-risk gene mutation was not significantly different between the two groups (42.3% vs. 40.0%, *p*-value = 0.885). The proportion of patients requiring transfusion was not significantly different between the two groups (88.5% vs. 73.3%, *p*-value = 0.390).

The association between TET2 mutation status and miR-22 expression was analyzed. The mean expression level of miR-22 was slightly higher in the mutant TET2 group compared with the WT TET2 group but was not statistically significant (3.077 vs. 2.183, *p*-value = 0.179, data not shown). Categorically, there was no significant association between TET2 mutation status and miR-22 expression status (*p*-value = 0.491, data not shown).

The association of miR-22 expression status with treatment outcomes of HMT was analyzed using the Chi-square test or Fisher’s exact test and the Kaplan–Meier method (Table 4). The rate of any response to HMT was 80.8% (21/26) in the low miR-22 expression group and 86.7% (13/15) in the high miR-22 expression group (*p*-value = 1.000). Specifically, both erythroid and platelet response rates were not significantly different between the two groups (*p*-values = 0.317 and 0.730, respectively). The disease progression rate was not significantly different between the two groups (61.5% vs. 40.0%, *p*-value = 0.183). The median time to progression was also not significantly different between the two groups (23.1 vs. 25.9 months, *p*-value = 0.155). Leukemic transformation tended to occur less frequently in the high miR-22 expression group (26.7%, 4/15) than in the low miR-22 expression group (57.7%, 15/26), but not statistically significant (*p*-value = 0.055). With a median follow-up time of 28.3 months (range, 8.7 to 83.5 months) in the low miR-22 expression group and 27.1 months (range, 7.5 to 95.9 months) in the high miR-22 expression group, the mortality rate was 73.1% (19/26) in the low miR-22 expression group and 53.3% (8/15) in the high miR-22 expression group (*p*-value = 0.199).

### 3.3. Association of Clinical Factors with Responsiveness to Hypomethylating Therapy, Leukemic Transformation, and Overall Survival

The association of various clinical factors with responsiveness to HMT was assessed via univariate logistic regression analysis. The odds ratios between various clinical factors and responsiveness to HMT are listed in Table 5. Similar to the results obtained using the Chi-square test or Fisher’s exact test, neither TET2 mutation nor high miR-22 expression was associated with responsiveness to HMT (*p*-value = 0.909, 0.631, respectively). In addition, responsiveness to HMT was not associated with other clinical factors, such as IPSS and IPSS-R risk, cytogenetic risk, the presence of poor-risk gene mutation and age above 70 years.

The association of various clinical factors with leukemic transformation was also assessed using univariate logistic regression analysis. The odds ratios between various clinical factors and leukemic transformation are listed in Table 6. Leukemic transformation tended to occur less frequently in the mutant TET2 group than in the WT TET2 group (OR= 0.170; 95% CI, 0.031–0.924; *p*-value = 0.040). Leukemic transformation tended to occur less frequently in the high miR-22 expression group than in the low miR-22 expression group but was not statistically significant (OR = 0.267; 95% CI, 0.067–1.064; *p*-value = 0.061). Patients with higher-risk disease based on IPSS tended to develop leukemic transformation more often than those with lower-risk disease based on IPSS (OR = 7.367; 95% CI, 1.836–29.544; *p*-value = 0.005). Higher-risk disease based on IPSS-R was also associated with increased leukemic transformation compared to lower-risk disease based on IPSS-R (OR = 4.900; 95% CI, 1.282–18.725; *p* = 0.020). The presence of any cytogenetic abnormality did not significantly increase the risk of leukemic transformation (OR = 2.400; 95% CI, 0.654–8.811; *p* = 0.187). Meanwhile, higher-risk karyotype based on IPSS-R was significantly associated with increased leukemic transformation compared to lower-risk karyotype (OR = 5.833; 95% CI, 1.038–32.797; *p* = 0.045). In addition, the incidence of leukemic transformation was significantly higher in patients with poor-risk gene mutation (OR = 17.733; 95% CI, 3.619–86.885; *p*-value = 0.000). Response to HMT and age above 70 years did not affect the incidence of leukemic transformation (*p*-value = 0.532, 0.477, respectively).

The association of various clinical factors with OS was analyzed using univariate Cox proportional hazards regression analysis (Table 7). TET2 mutation was associated with a trend toward better OS but was not statistically significant (HR = 0.354; 95% CI, 0.121–1.039; *p*-value = 0.059). High miR-22 expression was not associated with better OS (HR = 0.661; 95% CI, 0.289–1.513; *p*-value = 0.327). Patients with higher-risk disease based on IPSS tended to live shorter than those with lower-risk disease based on IPSS (HR = 2.093; 95% CI, 0.972–4.507; *p*-value = 0.059). Higher-risk disease based on IPSS-R was also associated with shorter OS compared to lower-risk disease based on IPSS-R (HR = 4.310; 95% CI, 1.724–10.776; *p*-value = 0.002). The presence of any cytogenetic abnormality was not significantly associated with OS (HR = 1.454; 95% CI, 0.666–3.173; *p*-value = 0.348). Meanwhile, higher-risk karyotype was associated with a trend toward poor OS compared to lower-risk karyotype but was not statistically significant (HR = 2.136; 95% CI, 0.905–5.041; *p*-value = 0.083). In addition, the presence of poor-risk gene mutation was significantly associated with poor OS (HR = 2.366; 95% CI, 1.093–5.120; *p*-value = 0.029). Response to HMT and age above 70 years did not significantly affect OS (*p*-value = 0.128, 0.206, respectively).

**Figure 2:**
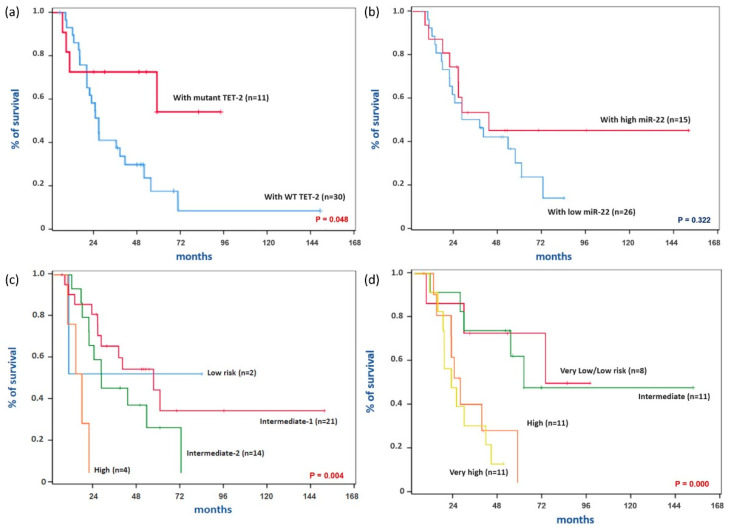
Kaplan–Meier survival curves according to various clinical factors. (**a**) Survival curves according to TET2 mutation status. (**b**) Survival curves according to miR-22 expression status. (**c**) Survival curves according to IPSS risk categories. (**d**) Survival curves according to IPSS-R risk categories. Abbreviations: TET2, Tet methylcytosine dioxygenase 2; IPSS, International Prognostic Scoring System; IPSS-R, Revised International Prognostic Scoring System. *p*-values were calculated using a log-rank test.

Kaplan–Meier survival curves according to various clinical factors were compared using a log-rank test (Figure 2). In this analysis, the mutant TET2 group showed significantly higher OS compared with the WT TET2 group (Figure 2a, *p*-value = 0.048). The estimated OS at 24 months was 55.2% (95% CI, 46.0–64.4%) in the WT TET2 group and 72.7% (95% CI, 59.3–86.1%) in the mutant TET2 group. OS was not significantly different between the high and low miR-22 expressing groups (Figure 2b, *p*-value = 0.322). The estimated OS at 24 months was 73.3% (95% CI, 61.9–84.7%) in the high miR-22 expression group and 56.0% (95% CI, 46.1–65.9%) in the low miR-22 expression group. Consistent with the results of univariate Cox proportional hazards regression analysis, the differences in OS according to IPSS and IPSS-R risk categories were statistically significant (Figure 2c,d, *p*-value = 0.004, 0.000, respectively). Interestingly, the survival curves according to TET2 mutation status showed a pattern similar to those of risk groups based on IPSS and IPSS-R. Therefore, the strength of correlation between TET2 mutation status and risk groups based on IPSS and IPSS-R was analyzed using Goodman–Kruskal’s gamma (data not shown). As expected, TET2 mutation was strongly correlated with higher-risk disease based on IPSS (Gamma = −0.674, *p*-value = 0.027) and IPSS-R (Gamma = −0.800, *p*-value = 0.003). Meanwhile, high miR-22 expression was not significantly correlated with higher-risk disease based on IPSS (Gamma = −0.525, *p*-value = 0.074) and IPSS-R (Gamma = −0.412, *p*-value = 0.177).

## 4. Discussion

We conducted this study to determine whether TET2 mutation and miR-22 expression represent biomarkers for predicting response to HMT. Since the response to HMT occurs after the fourth to sixth cycle of treatment and is achieved in only about 50% of patients with MDS [6], biomarkers for predicting response to HMT are needed. In our study, the response rate to HMT was not significantly different between the WT and mutant TET2 groups. Similarly, the response rate to HMT was not significantly different between the low and high miR-22 expression groups.

It is still debated whether TET2 mutation can serve as a marker for predicting response to HMT. Some studies reported that TET2 mutation was a predictive marker of response to HMT. Itzykson et al. first reported that the response rate to azacitidine was higher in the mutant TET2 group than in the WT TET2 group [14]. In the study of Bejar et al., mutant TET2 patients showed only a trend toward increased responsiveness to HMT compared with WT TET2 patients [15]. However, in many other studies, TET2 mutation status did not affect responsiveness to HMT in patients with MDS and other related neoplasms [22,23,24]. In the case of miR-22, no study has reported the relationship between miR-22 expression status and responsiveness to HMT.

This discrepancy may be due to the heterogeneity of DNA methylation patterns among patients with MDS regardless of TET2 mutation. Reilly et al. reported that MDS patients could be categorized into five distinct clusters according to DNA methylation patterns [25]. In this study, patients with TET2 mutations were classified into clusters B, C and E but enriched only in cluster D. Interestingly, the DNA methylation pattern was not significantly different between TET2 mutants and non-mutants within a single cluster. Furthermore, the comparison of TET2 mutants within a single cluster with TET2 mutants in other clusters revealed differences in methylation, which were consistent with global differences between clusters. These results suggest that TET2-mutant patients can diverge to different epigenetic states, potentially driven by patterns of coincident mutations or other factors, which may affect the responsiveness to HMT together with TET2 mutation.

Interestingly, in our study, patients in the mutant TET2 group had lower-risk disease than those in the WT TET2 group. Moreover, patients in the mutant TET2 group showed less leukemic transformation and better overall survival than those in the WT TET2 group. These results may be associated with chromosomal stability affected by DNA methylation. DNA methylation is known to play a key role in stabilizing heterochromatin by repressing translocations induced by the transcription of endogenous transposons and avoiding guanine quadruplexes-induced DNA breaks [4]. In our study, cytogenetic abnormality tended to be less frequent in the mutant TET2 group than in the WT TET2 group (18.2% vs. 43.3%, *p*-value = 0.168). Similarly, in the study of Bejar et al., patients in the mutant TET2 group tended to manifest fewer cytogenetic abnormalities and lower-risk disease than those in the WT TET2 group [15]. Itzykson et al. also reported that karyotype according to IPSS was more favorable in TET2-mutated patients compared with WT-TET2 patients, but IPSS risk was not significantly different between the two groups [14]. In addition, in the study of Smith et al., there was a limited tendency toward poor karyotype and higher-risk disease in the WT TET2 group compared with the mutant TET2 group but was not statistically significant [26]. Meanwhile, in our study, the frequency of cytogenetic abnormalities was not significantly different between the low and high miR-22 expression groups. Furthermore, although there was a tendency for high miR-22 expression to be associated with lower-risk disease based on IPSS, lower leukemic transformation and longer survival, this tendency was weak compared with that of mutant TET2.

It is also unclear whether the loss of TET2 affects leukemic transformation and survival in patients with MDS. Shiozawa et al. reported that TET2 mutation was enriched in the EMK subgroup, which showed better survival and lower leukemic transformation compared with the IMP subgroup [27]. However, in this study, TET2 mutation was not unique to the EMK subgroup but was slightly detected in the IMP subgroup. Reilly et al. also reported that TET2 mutation was enriched in, but not unique to, cluster D, which belongs to the low cluster risk group with better survival than the high cluster risk group [25]. However, Smith et al. reported no significant differences in leukemic transformation and survival between mutant and WT TET2 groups [26]. In addition, in other studies, there was no significant difference in survival between the mutant and WT TET2 groups [14,15,22,23]. Similar to HMT responsiveness, concurrent mutations or other factors may influence leukemic transformation and survival together with TET2 mutation.

The study has some limitations. First, the overall response rate of the entire cohort to HMT was nearly 80%, which is higher than the common response rate of 50% [6]. This high response rate may be attributed to the small size of the entire cohort. Second, the presence of TET2 mutation and high miR-22 expression cannot guarantee loss of TET2 function. In our analysis, it was not distinguished whether TET2 mutation is heterozygous or homozygous due to the small number of patients having TET2 mutation. Fortunately, Ko et al. reported that the global levels of 5hmC were significantly reduced in patients carrying either homozygous or heterozygous TET2 mutations compared with patients bearing WT TET2 [28]. This means that even if the TET2 mutation is heterozygous, it is in a sufficient loss-of-function state. However, unfortunately, we cannot be sure that there is no difference between the heterozygous and homozygous TET2 mutation. Indeed, heterozygous TET2 knockout in mice, which led to ~50% loss of TET2 gene expression, resulted in significant but slower and less frequent malignant transformation than double-allele knockout [29]. Moreover, AML patients with homozygous TET2 mutation showed significantly inferior event-free survival and a higher relapse rate compared with those with heterozygous TET2 mutation [30]. Meanwhile, in the case of miR-22 expression, Song et al. reported that some patients manifested both high levels of miR-22 and TET2 simultaneously [13]. Unfortunately, the expression of TET2 was not analyzed in our study. This may be why there was no difference in the frequency of cytogenetic abnormalities between the low and high miR-22 expression groups, and the tendency of high miR-22 expression to have lower-risk disease based on IPSS, lower leukemic transformation and longer survival was weaker than that of mutant TET-2.

In conclusion, TET2 mutation is not a good biomarker for predicting response to HMT. The miR-22 expression level is also not a good biomarker for predicting response to HMT. Responsiveness to HMT is thought to be affected by many other factors together with loss of TET2 function. Loss of TET2 function, including both TET2 mutation and high miR-22 expression, may be associated with lower-risk disease based on IPSS, lower leukemic transformation and longer survival. This may be associated with chromosomal stability affected by DNA methylation, which may contribute to the low frequency of cytogenetic abnormality in patients with mutant TET2 compared with those carrying WT TET2.

## Figures and Tables

**Table 1:** Baseline characteristics according to TET2 mutation status.

	WT TET2 (*n* = 30)	Mutant TET2 (*n* = 11)	*p*-Value
Median age	63 (23–86)	65 (38–82)	0.835
Sex (M/F)	18/12	8/3	0.716
Disease subtypes			0.434
MDS-SLD	2 (6.7)	0 (0.0)	
MDS-MLD	6 (20.0)	4 (36.4)	
MDS-EB1	9 (30.0)	2 (18.2)	
MDS-EB2	8 (26.7)	3 (27.3)	
MDS-U	1 (3.3)	0 (0.0)	
CMML	4 (13.3)	1 (9.1)	
5q-syndrome	0 (0.0)	1 (9.1)	
IPSS risk categories Low Intermediate-1 Intermediate-2 High Lower-risk disease * Higher-risk disease *	1 (3.3) 13 (43.3) 12 (40.0) 4 (13.3) 14 (46.7) 16 (53.3)	1 (9.1) 8 (72.7) 2 (18.2) 0 (0.0) 9 (81.8) 2 (18.2)	**0.039** 0.075
IPSS-R risk categories Very low Low Intermediate High Very high Lower-risk group * Higher-risk group *	1 (3.3) 3 (10.0) 6 (20.0) 9 (30.0) 11 (36.7) 10 (33.3) 20 (66.7)	0 (0.0) 4 (36.4) 5 (45.5) 2 (18.2) 0 (0.0) 9 (81.8) 2 (18.2)	**0.009** **0.006**
Cytogenetic abnormality	13 (43.3)	2 (18.2)	0.168
Cytogenetic risk categories			
Very good	2 (6.7)	0 (0.0)	0.102
Good	19 (63.3)	11 (100.0)	
Intermediate	2 (6.7)	0 (0.0)	
Poor	2 (6.7)	0 (0.0)	
Very poor	5 (16.7)	0 (0.0)	
Lower-risk karyotype ◊	21 (70.0)	11 (100.0)	0.083
Higher-risk karyotype ◊	9 (30.0)	0 (0.0)	
Poor-risk gene mutation	13 (43.3)	4 (36.4)	0.736
Transfusion requirement	26 (86.7)	8 (72.7)	0.361

Abbreviations: CMML, chronic myelomonocytic leukemia; IPSS, International Prognostic Scoring System; IPSS-R, Revised International Prognostic Scoring System; MDS-SLD, MDS-single lineage dysplasia; MDS-MLD, MDS-multilineage dysplasia; MDS-EB1, MDS-excess blasts-1; MDS-EB2, MDS-excess blasts-2; MDS-U, MDS-unclassified; TET2, Tet methylcytosine dioxygenase 2; WT, wild-type. * Based on the IPSS and IPSS-R, patients are divided into lower-risk disease (IPSS low, intermediate-1; IPSS-R very low, low, intermediate) and higher-risk disease (IPSS Intermediate-2, high; IPSS-R high, very high) groups. ◊ Based on the IPSS-R, cytogenetic risk categories are divided into lower-risk karyotype (IPSS-R very good, good) and higher-risk karyotype (IPSS-R intermediate, poor, very poor).

**Table 2:** Treatment outcomes of HMT according to TET2 mutation status.

Variables	WT TET2 (*n* = 30)	Mutant TET2 (*n* = 11)	*p*-Value
Any response to HMT	25 (83.3)	9 (81.8)	1.000
Hematologic improvement			
- Erythroid	20 (66.7)	6 (54.5)	0.491
- Platelet	21 (70.0)	5 (45.5)	0.272
Disease progression	19 (63.3)	3 (27.3)	**0.040**
Median time to progression (m) *	19.3 (5.5–47.8)	49.8 (6.7–95.9)	**0.007**
Leukemic transformation	17 (56.7)	2 (18.2)	**0.029**
Death	23 (76.7)	4 (36.4)	**0.026**

Abbreviations: HMT, hypomethylating therapy; m, month; TET2, Tet methylcytosine dioxygenase 2; WT, wild-type. * Median time to progression was calculated with Kaplan–Meier method using intention-to-treat (ITT) analysis. A log-rank test was used to compare TTP.

**Table 3:** Baseline characteristics according to miR-22 expression status.

	Low miR-22 Expression (*n* = 26)	High miR-22 Expression (*n* = 15)	*p*-Value
Median age	63 (42–86)	60 (23–79)	0.422
Sex (M/F)	16/10	10/5	0.743
Disease subtypes			**0.061**
MDS-SLD	1 (3.8)	1 (6.7)	
MDS-MLD	4 (15.4)	6 (40.0)	
MDS-EB1	5 (19.2)	6 (40.0)	
MDS-EB2	9 (34.6)	2 (13.3)	
MDS-U	1 (3.8)	0 (0.0)	
CMML	5 (19.2)	0 (0.0)	
5q-syndrome	1 (3.8)	0 (0.0)	
IPSS risk categories Low Intermediate-1 Intermediate-2 High Lower-risk disease * Higher-risk disease *	2 (7.7) 10 (38.5) 10 (38.5) 4 (15.4) 12 (46.2) 14 (53.8)	0 (0.0) 11 (73.3) 4 (26.7) 0 (0.0) 11 (73.3) 4 (26.7)	0.149 0.091
IPSS-R risk categories			0.429
Very low	1 (3.8)	0 (0.0)	
Low	3 (11.5)	4 (26.7)	
Intermediate	6 (23.1)	5 (33.3)	
High	9 (34.6)	2 (13.3)	
Very high	7 (26.9)	4 (26.7)	0.183
Lower-risk disease *	10 (38.5)	9 (60.0)	
Higher-risk disease *	16 (61.5)	6 (40.0)	
Cytogenetic abnormality	9 (34.6)	6 (40.0)	0.730
Cytogenetic risk categories			
Very good	1 (3.8)	1 (6.7)	0.757
Good	20 (76.9)	10 (66.7)	
Intermediate	1 (3.8)	1 (6.7)	
Poor	1 (3.8)	1 (6.7)	
Very poor	3 (11.5)	2 (13.3)	
Lower-risk karyotype ◊	21 (80.8)	11 (73.3)	0.701
Higher-risk karyotype ◊	5 (19.2)	4 (26.7)	
Poor-risk gene mutation	11 (42.3)	6 (40.0)	0.885
Transfusion requirement	23 (88.5)	11 (73.3)	0.390

Abbreviations: CMML, chronic myelomonocytic leukemia; IPSS, International Prognostic Scoring System; IPSS-R, Revised International Prognostic Scoring System; MDS-SLD, MDS-single lineage dysplasia; MDS-MLD, MDS-multilineage dysplasia; MDS-EB1, MDS-excess blasts-1; MDS-EB2, MDS-excess blasts-2; MDS-U, MDS-unclassified. * Based on IPSS and IPSS-R, patients are divided into lower-risk disease (IPSS low, intermediate-1; IPSS-R very low, low, intermediate) and higher-risk disease (IPSS Intermediate-2, high; IPSS-R high, very high) groups. ◊ Based on the IPSS-R, cytogenetic risk categories are divided into lower-risk karyotype (IPSS-R very good, good) and higher-risk karyotype (IPSS-R intermediate, poor, very poor).

**Table 4:** Treatment outcomes of HMT according to miR-22 expression status.

Variables	Low miR-22 Expression (*n* = 26)	High miR-22 Expression (*n* = 15)	*p*-Value
Any response to HMT	21 (80.8)	13 (86.7)	1.000
Hematologic improvement			
- Erythroid	15 (57.7)	11 (73.3)	0.317
- Platelet	17 (65.4)	9 (60.0)	0.730
Disease progression	16 (61.5)	6 (40.0)	0.183
Median time to progression (m) *	23.1 (5.5–67.1)	25.9 (6.7–95.9)	0.155
Leukemic transformation	15 (57.7)	4 (26.7)	**0.055**
Death	19 (73.1)	8 (53.3)	0.199

Abbreviations: HMT, hypomethylating therapy; m, month. * Median time to progression was calculated with the Kaplan–Meier method using intention-to-treat (ITT) analysis. A log-rank test was used to compare TTP.

**Table 5:** Association of various clinical factors with overall response to hypomethylating therapy.

Variables	Any Response to HMT	
	Odds Ratio (95% CI)	*p*-Value ^a^
TET2 mutation	0.900 (0.148–5.489)	0.909
High miR-22 expression	1.548 (0.261–9.175)	0.631
IPSS (higher-risk to lower-risk disease) ^b^	2.222 (0.377–13.082)	0.377
IPSS-R (higher-risk to lower-risk disease) ^b^	1.689 (0.327–8.732)	0.532
Cytogenetic abnormality	4.200 (0.454–38.843)	0.206
Cytogenetic risk categories	1.846 (0.193–17.700)	0.595
(higher-risk to lower-risk karyotype) ^c^		
Poor-risk gene mutation	0.933 (0.180–4.838)	0.935
Elderly (≥70)	0.933 (0.180–4.838)	0.935

Abbreviations: CI, confidence interval; HMT, hypomethylating therapy; IPSS, International Prognostic Scoring System; IPSS-R, Revised International Prognostic Scoring System; TET2, Tet methylcytosine dioxygenase 2. ^a^ *p*-value was calculated via univariate logistic regression analysis. ^b^ Based on the IPSS and IPSS-R, patients are divided into lower-risk disease (IPSS low, intermediate-1; IPSS-R very low, low, intermediate) and higher-risk disease (IPSS Intermediate-2, high; IPSS-R high, very high) groups. ^c^ Based on the IPSS-R, cytogenetic risk categories are divided into lower-risk karyotype (IPSS-R very good, good) and higher-risk karyotype (IPSS-R intermediate, poor, very poor).

**Table 6:** Association of various clinical factors with leukemic transformation.

Variables	Leukemic Transformation	
	Odds Ratio (95% CI)	*p*-Value ^a^
TET2 mutation	0.170 (0.031–0.924)	**0.040**
High miR-22 expression	0.267 (0.067–1.064)	**0.061**
IPSS (higher-risk to lower-risk disease) ^b^	7.367 (1.836–29.554)	**0.005**
IPSS-R (higher-risk to lower-risk disease) ^b^	4.900 (1.282–18.725)	**0.020**
Cytogenetic abnormality	2.400 (0.654–8.811)	0.187
Cytogenetic risk categories	5.833 (1.038–32.797)	**0.045**
(higher-risk vs. lower-risk karyotype) ^c^		
Poor-risk gene mutation	17.733 (3.619–86.885)	**0.000**
Response to HMT	0.592 (0.115–3.061)	0.532
Elderly (≥70)	1.575 (0.451–5.504)	0.477

Abbreviations: CI, confidence interval; HMT, hypomethylating therapy; IPSS, International Prognostic Scoring System; IPSS-R, Revised International Prognostic Scoring System; TET2, Tet methylcytosine dioxygenase 2. ^a^ *p*-value was calculated via univariate logistic regression analysis. ^b^ Based on the IPSS and IPSS-R, patients are divided into lower-risk disease (IPSS low, intermediate-1; IPSS-R very low, low, intermediate) and higher-risk disease (IPSS Intermediate-2, high; IPSS-R high, very high) groups. ^c^ Based on the IPSS-R, cytogenetic risk categories are divided into lower-risk karyotype (IPSS-R very good, good) and higher-risk karyotype (IPSS-R intermediate, poor, very poor).

**Table 7:** Association of various clinical factors with overall survival.

Variables	Overall Survival	
	Hazard Ratio (95% CI)	*p*-Value ^a^
TET2 mutation	0.354 (0.121–1.039)	**0.059**
High miR-22 expression	0.661 (0.289–1.513)	0.327
IPSS (higher-risk vs. lower-risk disease) ^b^	2.093 (0.972–4.507)	**0.059**
IPSS-R (higher-risk vs. lower-risk disease) ^b^	4.310 (1.724–10.776)	**0.002**
Cytogenetic abnormality	1.454 (0.666–3.173)	0.348
Cytogenetic risk categories	2.136 (0.905–5.041)	0.083
(higher-risk vs. lower-risk karyotype) ^c^		
Poor-risk gene mutation	2.366 (1.093–5.120)	**0.029**
Response to HMT	0.490 (0.196–1.228)	0.128
Elderly (≥70)	1.639 (0.762–3.525)	0.206

Abbreviations: CI, confidence interval; HMT, hypomethylating therapy; IPSS, International Prognostic Scoring System; IPSS-R, Revised International Prognostic Scoring System; TET2, Tet methylcytosine dioxygenase 2. ^a^ *p*-value was calculated via univariate Cox proportional hazards regression analysis. ^b^ Based on the IPSS and IPSS-R, patients are divided into lower-risk disease (IPSS low, intermediate-1; IPSS-R very low, low, intermediate) and higher-risk disease (IPSS Intermediate-2, high; IPSS-R high, very high) groups. ^c^ Based on the IPSS-R, cytogenetic risk categories are divided into lower-risk karyotype (IPSS-R very good, good) and higher-risk karyotype (IPSS-R intermediate, poor, very poor).
